# RNA sequencing-based longitudinal transcriptomic profiling gives novel insights into the disease mechanism of generalized pustular psoriasis

**DOI:** 10.1186/s12920-018-0369-3

**Published:** 2018-06-05

**Authors:** Lingyan Wang, Xiaoling Yu, Chao Wu, Teng Zhu, Wenming Wang, Xiaofeng Zheng, Hongzhong Jin

**Affiliations:** Department of Dermatology, Peking Union Medical College Hospital, Chinese Academy of Medical Sciences and Peking Union Medical College, Beijing, China

**Keywords:** Generalized pustular psoriasis, RNA-sequencing, Neutrophil, Peripheral blood mononuclear cell

## Abstract

**Background:**

Generalized pustular psoriasis (GPP) is a rare, episodic, potentially life-threatening inflammatory disease. However, the pathogenesis of GPP, and universally accepted therapies for treating it, remain undefined.

**Methods:**

To better understand the disease mechanism of GPP, we performed a transcriptome analysis to profile the gene expression of peripheral blood mononuclear cells (PBMCs) from patients enrolled at the time of diagnosis and receiving follow-up treatment for up to 6 months.

**Results:**

RNA sequencing data revealed that gene expression in five GPP patients’ PBMCs was profoundly altered following acitretin treatment. Differentially expressed gene (DEG) analysis suggested that genes related to psoriatic inflammation, including *CXCL1*, *CXCL8 (IL-8)*, *S100A8, S100A9, S100A12* and *LCN2*, were significantly downregulated in patients in remission from GPP. Functional enrichment and annotation analysis unveiled a cluster of DEGs significantly associated with the function of leukocytes, particularly neutrophils. Pathway analysis suggested that a variety of pro-inflammatory pathways were inhibited in patients in remission. This analysis not only reaffirmed known signaling pathways in GPP pathogenesis, but also implicated novel factors and pathways, such as cell cycle regulation pathways. Furthermore, regulator network analysis provided bioinformatics-based support for upstream molecules as potential therapeutic targets such as oncostatin M.

**Conclusions:**

This longitudinal analysis of blood transcriptomes provides the first evidence that dysregulated gene expression in peripheral blood may significantly contribute to psoriatic inflammation in GPP patients. Novel canonical pathways and biomarkers identified in the current research may provide insights to help understand GPP pathobiology and advance novel therapeutics.

**Electronic supplementary material:**

The online version of this article (10.1186/s12920-018-0369-3) contains supplementary material, which is available to authorized users.

## Background

Generalized pustular psoriasis (GPP) is a potentially life-threatening, multisystemic inflammatory disease characterized by sudden, repeated episodes of high-grade fever, generalized erythematous pustular rashes, and systemic upset. GPP is often triggered by environmental factors and immune disorders, such as pregnancy, infections, drugs and hypocalcemia [1]. Although GPP has long been considered to be a rare variant of psoriasis, some evidence suggests that it is a different entity: different clinical presentation and distinct HLA alleles have been observed in patients with GPP compared with common forms of psoriasis [2–4]. To date, the pathogenesis and pathophysiology of GPP remain largely unknown. Dysregulated expression of cytokines/chemokines, such as IL-1 and IL-36, have been suggested to play important roles in GPP [5]. In the last few years, rare variants of the genes, *IL36RN*, *CARD14* and *AP1S3* have been identified as susceptibility factors for GPP [6–8]. Nevertheless, many GPP patients do not carry mutations in any of these three genes, leaving the genetic basis of GPP elusive [9].

GPP is a difficult disease to treat. Therapies successful for treating plaque psoriasis are generally less effective for GPP. Since 2012, acitretin, cyclosporine or methotrexate have been the recommended first-line therapies for acute GPP. Of these, acitretin, an oral retinoid, is the preferred agent [10]. Acitretin has shown success in treating both generalized and localized pustular psoriasis, while it is less effective for plaque psoriasis [11]. Ozawa et al. [12] demonstrated that the oral retinoid has higher effectiveness in GPP patients than methotrexate, cyclosporine, psoralen and ultraviolet A irradiation. Nevertheless, the mechanism of action of acitretin still remains largely unclear, impeding its broader application. Moreover, some GPP patients do not respond to existing treatments, creating an urgent need for novel drug targets and therapeutics.

Transcriptome profiling technologies, such as microarrays and RNA sequencing (RNA-seq), are valuable tools for deciphering the regulatory network underlying disease. Recently, by performing microarray analysis with skin lesions from GPP patients, researchers have successfully identified critical genes or pathways in GPP [13, 14]. To better understand GPP pathogenesis and drug effects at the molecular level, we performed an RNA-seq-based longitudinal gene expression study of peripheral blood mononuclear cells (PBMCs) obtained from GPP patients before and during acitretin treatment. Differentially expressed genes were systematically identified and further analyzed by functional network annotation. Our study comprehensively profiled the molecular signature of GPP patients in response to drug treatment, and provides clues for potential new drug targets for GPP treatment.

## Methods

### Patient enrollment and sampling

This study was approved by the Medical Ethics Committee of the Peking Union Medical College Hospital. Five adult patients with GPP who responded well to acitretin treatment were included. All patients were diagnosed according to the Umezawa criteria and presented with clinically visible generalized pustules at their initial visit [15]. All patients had not undergone any systemic treatment for at least 1 month.

After receipt of written informed consent signed by the patients, 10 ml whole-blood samples were obtained from the patients at T0, T1 and T2. Blood was collected in endotoxin-free silicone-coated tubes. The PBMCs were prepared with Ficoll-Paque PLUS (GE Healthcare, Uppsala, Sweden) according to the manufacturer’s instructions. The PBMCs obtained from each sample were stored at − 80 °C in sterile screw-cap tubes and thawed directly before analysis. IL36RN mutations of all the patients were detected by using the previously described methods [16].

### RNA isolation and sequencing library construction

Total RNA was extracted using Trizol (Invitrogen, Carlsbad, CA) according to the manufacturer’s instructions. RNA purity was checked using a kaiaoK5500® spectrophotometer (Kaiao, Beijing, China), and its integrity and concentration were assessed using an RNA Nano 6000 Assay Kit with a Bioanalyzer 2100 system (Agilent Technologies, CA). Total RNA meeting the following conditions was used for library construction: the RNA integrity number (RIN) ≥ 7; 28S/18S rRNA ratio ≥ 1.5.

One microgram of total RNA per sample was used as initial material for library construction. Sequencing libraries with varied index labels were generated for each sample following the manufacturer’s recommendations, using an NEBNext® Ultra™ RNA Library Prep Kit (NEB, Ipswich, MA). The library construction procedures were as follows. First, ribosomal RNA was removed using an Illumina Ribo-Zero™ Gold rRNA Removal Kit. RNA fragmentation was then carried out. Next, the first and second cDNA strand were sequentially synthesized. The library fragments were then purified, followed by terminal repair, dA-tailing and adapter ligation. The library fragments were purified, and UNG enzyme digestion were performed. Finally, polymerase chain reaction amplification was carried out to complete the library construction.

### Library clustering and sequencing

Clustering of the index-coded samples was performed on a cBot cluster generation system using a TruSeq PE Cluster Kit v4-cBot-HS (Illumina, San Diego, CA) according to the manufacturer’s instructions. After cluster generation, the libraries were sequenced on an Illumina Hiseq X10 platform (Illumina, San Diego, CA), and 125 bp paired-end reads were generated by CapitalBioTech (Beijing, China).

### Quality control and read alignment

Quality control metrics were obtained for raw sequencing reads using the FastQC application [17]. Reads were mapped to the hg19 human reference genome with TopHat2 and mapped read counts were estimated by Cufflinks [18]. Transcript expression was calculated from values for fragments per kilobase of exon per million fragments mapped.

### DEG analysis and statistical analysis

Differential gene expression analysis was performed using the Limma package of R language (v. 3.22.7), an R/Bioconductor software package that provides an integrated solution for both differential expression and differential splicing analyses of RNA-seq data [19]. Paired t-tests were used to compare gene expression values between the pre- and the posttreatment samples (T1 versus T0 and T2 versus T0). The Benjamini–Hochberg method was used as an FDR adjustment for multiple testing correction. A threshold of FDR < 0.05 was used to define statistical significance. Venn diagrams showing the overlap of DEGs in different groups were constructed using an online bioinformatics tool (http://bioinformatics.psb.ugent.be/webtools/Venn/). Principle component analysis was conducted with ClustVis [20] (http://biit.cs.ut.ee/clustvis/).

### Functional analysis

Metascape (http://metascape.org) was employed to perform the gene enrichment and functional annotation analyses. For the identification of pathways and regulatory networks, Canonical Pathway Analysis, Upstream Regulator Analysis, and Regulatory Effects Analysis were performed using the IPA software (Ingenuity Systems, Redwood City, CA, USA; Version: 42012434).

### Reverse transcription–qPCR (RT-qPCR)

RT-qPCR was performed on 10 preselected GPP-related genes. RNA was extracted using Trizol (Invitrogen, Carlsbad, CA, USA). The sequences of the qPCR primers used in this study are shown in Additional file 1: Table S1. Each RT-qPCR reaction was performed in duplicate and the mean threshold cycle (Ct) value for each sample was used for data analysis. The 2^−ΔΔCt^ method was used for determining the fold-change in the expression level, and *GAPDH* was used for normalization. Paired t-test analyses were performed on the 2^−ΔΔCt^ values for the comparison of two groups of samples.

## Results

### Patient sample collection, sequencing data and differential expression analysis

Five adult patients affected with GPP were included in the current study (one male and four females, aged 36.40 ± 18.72 years). All patients fulfilled the diagnostic criteria for GPP at their initial visit [15]. Treatment was started with acitretin on an initial dose of 0.5–0.75 mg/kg/day. All five patients achieved remission of their clinical symptoms after treatment for two weeks: pustules cleared, body temperature recovered and general symptoms improved. Once pustulation was resolved, acitretin was tapered down gradually to a final dose of 10 mg/day for long-term maintenance. Disease severity of the patients was quantified using the GPP severity score system [21]. The characteristics of these five patients are shown in Table 1.

**Table 1:** Characteristics of generalized pustular psoriasis patients

Patient	AoO^a^/Duration (yr)	Age range (yr)	PsA^b^ [43]	PsV^c^	GPP score			*IL36RN* Mutation
					T0	T1	T2	
P1	21 /13	30–39	–	–	12	5	2	c.115 + 6 T > C
P2	64/3	60–69	+	+	15	7	3	c.115 + 6 T > C
P3	24/0	20–29	–	+	15	6	2	c.115 + 6 T > C and c.227C > T
P4	37/1	30–39	+	+	11	6	1	Not detected
P5	19/0	10–20	–	+	4	4	1	Not detected

^a^AoO = Age of Onset

^b^PsA = psoriatic arthritis

^c^PsV = psoriasis vulgari

**Fig. 1:**
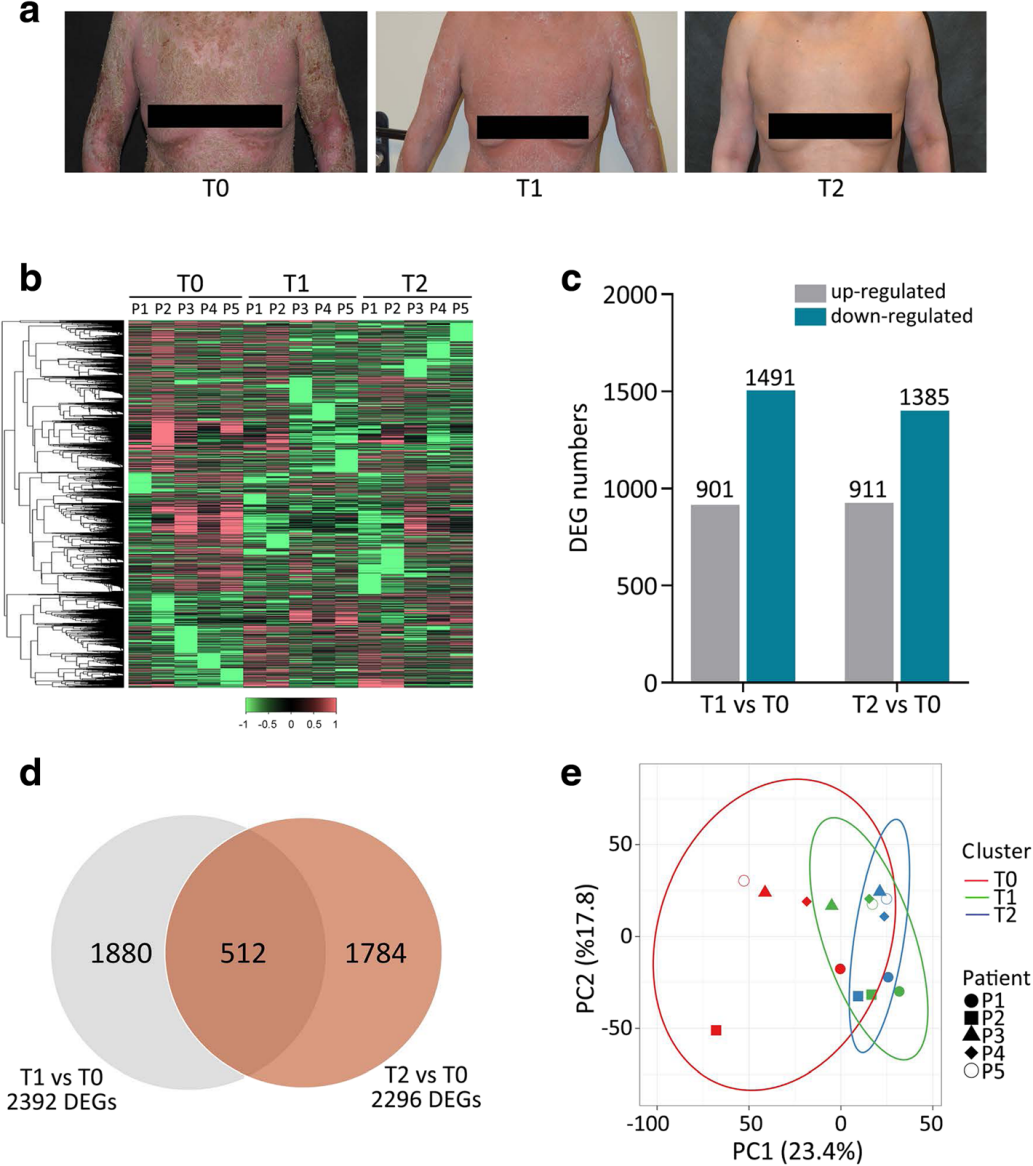
Differential gene expression analysis of GPP disease. **a** Clinical manifestation of a patient with GPP at the time of the acute phase (T0), 2 weeks after treatment (T1), and 6 months after treatment (T2). **b** Heatmap visualization of the z-scores for DEGs identified in PBMCs from five patients with GPP (P1–P5) at T0, T1 and T2. **c** Bar chart of the numbers of DEGs. **d** Venn diagram representing the numbers of DEGs in the T1 versus T0 and T2 versus T0 comparisons. **e** Principal component analysis. Projections of patients (P1–P5) at different stages (T0–T2) described by all significant DEGs for the first two principal components (PC1, PC2)

To investigate the pathophysiological mechanisms underlying remission of GPP, transcriptome profiling was performed with PBMC samples collected at three time points: T0 (the acute phase of GPP, prior to initiation of acitretin treatment), T1 (2 weeks after treatment) and T2 (6 months after treatment). At T1, all five patients showed clearance of pustules, which is the hallmark of the end of acute psoriatic inflammation. At T2, all five patients reached stable remission. The cutaneous manifestation of one GPP patient at the different time points of sample collection is shown in Fig. 1a.

RNA-seq was performed with each sample, and approximately 80 million reads were generated per sample. Sequencing reads were aligned to the human genome and analyzed as described in the methods. Differentially expressed coding genes (DEGs) were then identified by comparing the post-treatment with the pre-treatment transcriptomes of each patient (T1 versus T0 and T2 versus T0). An adjusted *p*-value (FDR < 0.05) and fold change (FC) ratio (|log2FC| ≥ 1) were used to determine the DEGs. A heatmap of DEGs shows global transcriptome changes in individual patients after receiving treatment (Fig. 1b). A total of 2392 DEGs were identified when comparing T1 with T0 samples. Of these, 901 were upregulated (38%) and 1491 downregulated (62%). A total of 2296 DEGs were identified from the comparison of T2 and T0 samples, with approximately 40% (*n* = 911) upregulated and 60% (*n* = 1385) downregulated (Fig. 1c). As shown in Fig. 1d, 512 DEGs were common to both the T1 versus T0 and T2 versus T0 datasets (Fig. 1d). The DEGs were sorted by statistical significance (lowest false discovery rate, FDR); the top 50 upregulated and downregulated DEGs in the T1 versus T0 and T2 versus T0 datasets are listed in Additional file 2: Table S2 and Additional file 3: Table S3, respectively. Next, principal component analysis (PCA) of the DEGs was conducted. We observed that the T0, T1 and T2 clusters partially overlapped, but the pre-treatment cluster (T0) was much more widely dispersed with less overlap than the two post-treatment clusters (T1 and T2) (Fig. 1e).

### Functional enrichment and annotation

**Fig. 2:**
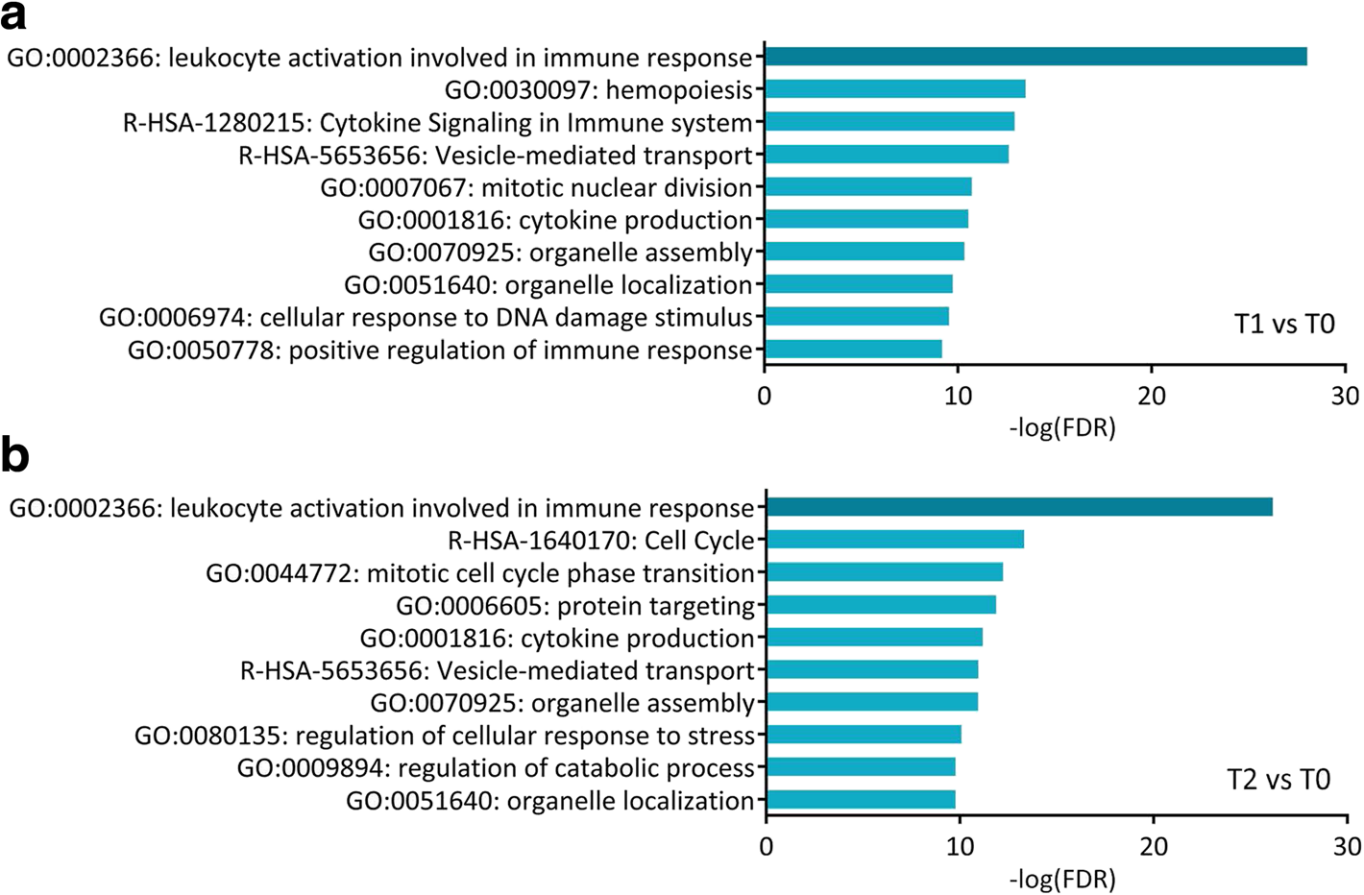
Functional enrichment and annotation for DEGs. Enrichment of the top 10 clusters at **a** T1 and **b** T2 was performed using Metascape analysis, which was carried out with the four GO sources: KEGG Pathway, GO Biological Processes, Reactome Gene Sets and CORUM. *p*-Values were calculated based on the accumulative hypergeometric distribution

Functional enrichment and annotation of DEGs were performed with Metascape [22] (http://metascape.org). Metascape analysis is carried out with four gene ontology (GO) sources: GO Biological Processes, Reactome Gene Sets, Kyoto Encyclopedia of Genes and Genomes (KEGG) Pathway, and the Comprehensive Resource of Mammalian protein complexes (CORUM). This analysis revealed that “leukocyte activation involved in immune response” was the most significantly enriched functional cluster identified at both T1 (−log(FDR) = 28.0) and T2 (−log(FDR) = 26.2) (Fig. 2a and b). Considering that GPP is a severe autoinflammatory disease with systemic inflammation, this result suggests that the remission of GPP is significantly associated with functional changes in peripheral blood leukocytes. DEGs were also enriched in several other clusters related to immune responses, including “cytokine signaling in immune system” (T1, −log(FDR) = 12.9), “cytokine production” (T1, −log(FDR) = 10.5; T2, −log(FDR) = 11.2) and “positive regulation of immune response” (T1, −log(FDR) = 9.2). Several clusters associated with cellular organelle biogenesis, including “vesicle-mediated transport” (T1, −log(FDR) = 12.6; T2, −log(FDR) = 11.0), “organelle assembly” (T1, −log(FDR) = 10.3; T2, −log(FDR) = 11.0) and “organelle localization” (T1, −log(FDR) = 9.7; T2, −log(FDR) = 9.7) were identified at both T1 and T2 (Fig. 2a and b).

To gain an in-depth understanding the relevance of circulating leukocytes in GPP, we further analyzed the DEGs in the “leukocyte activation involved in immune response” category. Metascape analysis revealed that these DEGs were significantly enriched in the category, “neutrophil activation involved in immune response” at both T1 (−log(FDR) = 96.8) and T2 (−log(FDR) = 96.8) (Additional file 4: Figure S1). Recently, a list has been assembled of genes known to be involved in particular aspects of neutrophil function [23]. By comparing our data with this list, we found that DEGs were identified in nearly every aspect of neutrophil biology (Table 2), suggesting that neutrophils actively function in the pathogenesis of GPP. Finally, a Molecular Complex Detection (MCODE) algorithm was further applied to identify densely connected networks in the leukocyte activation gene cluster. This analysis identified “regulated exocytosis”, a process involved in neutrophil activation, as the most significantly enriched MCODE component at both T1 (−logP = 20.0) and T2 (−logP = 16.0) (Additional file 5: Figure S2). Collectively, our data suggest that the GPP is associated with functional change of leukocytes, especially neutrophils, in the patients’ peripheral blood.

**Table 2:** Differential expressed genes in involved in neutrophil function

Process	Gene Symbol
Development	*CSF3R*
Protein trafficking	*CD63, AP3S1, AP3M2, AP1S3, AP1B1, AP1G2, AP1G1, AP2M1*
Granule formation	*MMP9, LTF, LCN2, HP, MMP8, DEFA1, DEFA4, ELANE, PRTN3, AZU1*
Capture and rolling	*PTX3*
Arrest	*ITGB2, CD44*
Activation during transmigration	*PIK3R5, MAPK14*
Pattern recognition, migration	*FPR1, FPR2, IFNGR1, LTB4R, TLR1, TLR5, MYD88, IRAK4, CLEC7A, NLRP3, NLRC4*
Cytokine secretion	*IL1B, IL8*
Immune crosstalk	*TNFSF13B, ARG1*
phagocytosis and degranulation	*NCF1, HCK, PTK2, RAB27A, RAB27B*
Pyroptosis, autophagy	*ATG3, GABARAPL1, CASP1*
Apoptosis	*HTRA2, CASP3*

### Pathway analysis

**Fig. 3:**
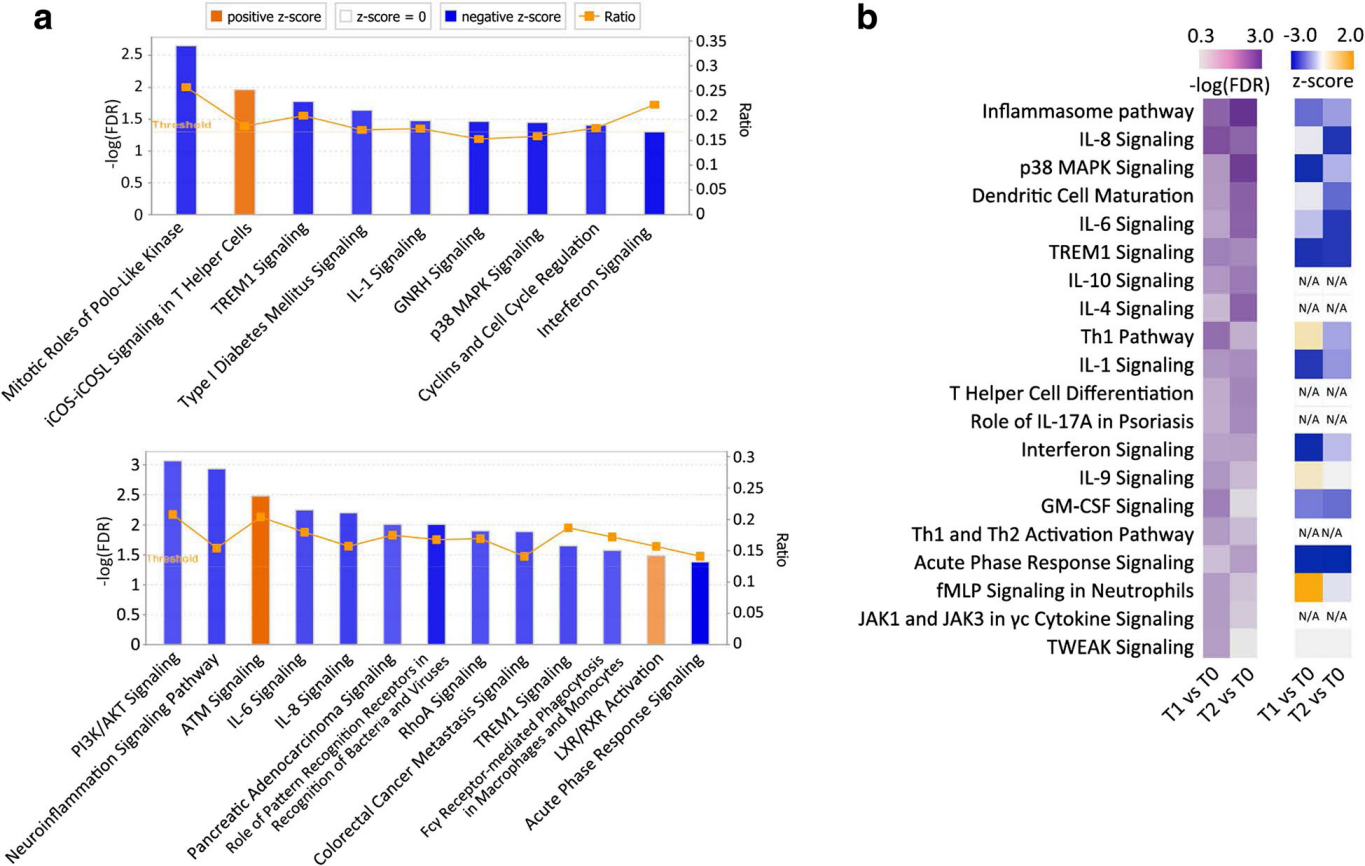
Pathway analysis. Enrichment of the top clusters was performed using IPA GO algorithms for all significant DEGs. **a** Enrichment of clusters for DEGs for T1 versus T0 (top) and T2 versus T0 (bottom). The threshold of minimum significance (−log(FDR)) was set to 1.3. The z-score predicts the direction of change for the function, and the threshold was set to an absolute value of z-score > 2. The height of the bars represents the probability that the DEGs in the dataset are associated with each pathway. The blue bars indicate predicted pathway activation, and orange indicate predicted inhibition. The orange points connected by a line represent the ratio which indicates the proportion of DEGs in the datasets that map to a given canonical pathway. **b** Canonical pathways associated with cytokine signaling. Enrichment of clusters associated with cytokine signaling was performed using IPA analysis (FDR < 0.05). The Benjamini–Hochberg method was used as an FDR adjustment for multiple testing correction. The top 20 enriched canonical pathways (ranked by average -log(FDR) value) are presented by showing the −log(FDR) and z-scores in heatmaps

To dissect signaling pathways involved in GPP pathogenesis, we performed Canonical Pathway Analysis with the Ingenuity Pathway Analysis (IPA) software. Based on IPA GO algorithms and KnowledgeBase mining, DEGs at T1 and T2 were enriched in 83 and 74 canonical pathways, respectively (FDR < 0.05, Benjamini-Hochberg adjusted *p*-value) (Additional file 6: Table S4). By using an absolute z-score value above 2 as a threshold, 9 pathways were identified at T1 (Fig. 3a, top), and 13 pathways were identified at T2 (Fig. 3a, bottom). Of note, most of the signaling pathways were predicted to be inhibited after acitretin treatment. The “TREM1 Signaling” pathway were found to be inhibited at both T1 (−log(FDR) = 1.78) and T2 (−log(FDR) = 1.75). The “Interferon Signaling” (−log(FDR) = 1.3) and “Role of Pattern Recognition Receptors in Recognition of Bacteria and Viruses” (−log(FDR) = 2.2) pathways were identified at T1 and T2, respectively (Additional file 7: Figure S3, Panels A–C). These pathways are mostly implicated in innate immune responses [24, 25]. In these pathways, the expression of innate immune response genes, such as *TLR1*, *TLR5*, *TLR8*, *MYD88*, *NLRC4*, *NOD2*, *IRF7*, *IFNAR1* and *STAT1* were significantly downregulated. Moreover, IPA analysis with all DEGs identified in the current study revealed that the number of innate immunity genes was substantially more than that of adaptive immunity genes (Additional file 8: Figure S4). These observations suggested that innate immune inflammation is predominantly involved in the inflammation seen in GPP patients. In addition, DEGs were found to be enriched in several cell cycle regulation pathways, including “Mitotic Roles of Polo-like Kinase” (−log(FDR) = 2.65) and “Cyclins and the Cell Cycle Regulation” (−log(FDR) = 1.41) (Additional file 9: Figure S5, Panels A and B), which is consistent with a previous finding showing dysregulated cell cycle gene expression in GPP patients [14].

Multiple cytokine signaling pathways that are known to play important roles in psoriasis pathogenesis were enriched in the DEGs, including the IL-1, IL-6 and IL-8 pathways (Fig. 3a). To comprehensively understand the regulation of cytokine signaling pathways in GPP, we performed a cytokine signaling-specific Canonical Pathway Analysis. The top 20 enriched cytokine signaling pathways (ranked by average –log(FDR) value) are presented by showing their –log(FDR) and z-score values in heatmap format (Fig. 3b). Judging from the z-score values, cytokine signaling pathways as a whole showed a generally more inhibitory pattern at T2 compared with T1, while the “Inflammasome pathway”, “p38 MAPK signaling”, “IL-1 signaling” and “Interferon signaling” pathways were less inhibited, suggesting that distinct cytokine signaling pathways may be regulated with different kinetics or to different extents in response to drug treatment. Notably, the “Role of IL-17A in Psoriasis” (T2, −log(FDR) = 1.67) pathway was enriched (Fig. 3b), in which downregulated *CXCL3*, *IL-8*, *S100A8*, *S100A9*, *IL17RA* and *CXCL1* expression was observed (Additional file 9: Figure S5, Panel C).

### Regulatory network analysis

**Fig. 4:**
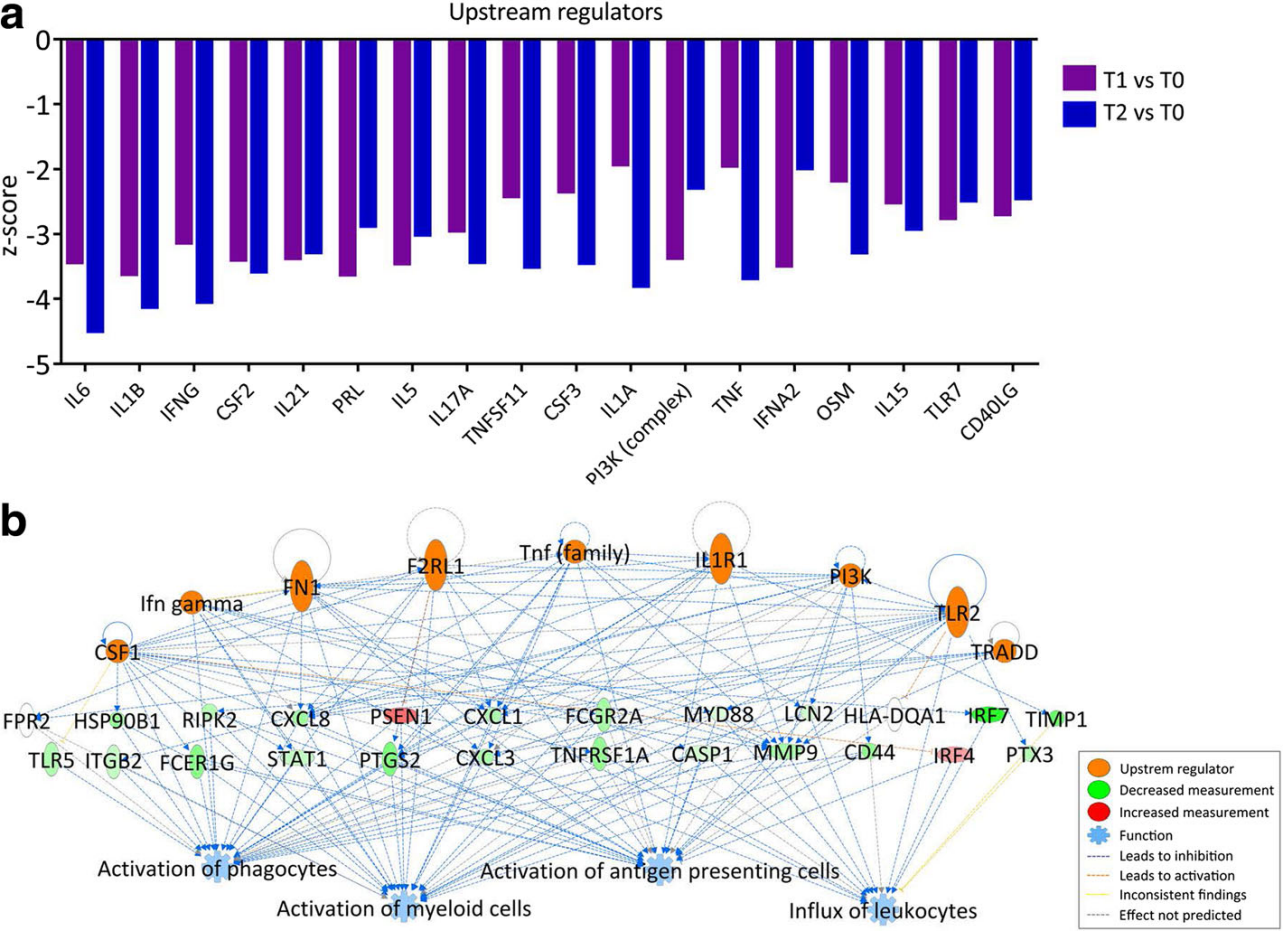
Regulatory network analysis. **a** Upstream Regulator Analysis. The top 20 upstream regulators associated with cytokine pathways (ranked by average z-score) are presented as a bar chart. The z-score is used to predict the activation state of a putative regulator (either activated or inhibited). **b** Regulatory Effects Analysis. The Regulatory Effects network was generated by connections between predicted upstream regulators, DEGs, and predicted downstream functions or diseases. The network with the highest consistency score is presented

Next, we performed regulatory network analysis to identify gene interactions and regulatory cascades, again using IPA software. First, Upstream Regulator Analysis, which is based on expected causal effects between upstream regulators and targets, was carried out to predict upstream molecules that may be responsible for gene expression changes. The upstream regulator candidates selected for this analysis were “cytokine” and “transmembrane receptor” because cytokine signaling plays a central role in GPP pathogenesis. The top 20 upstream regulators (*p* < 0.01), ranked by absolute z-score, are shown in Fig. 4a. Remarkably, all 20 upstream regulators have negative z-scores, indicating that their downstream effects were inhibited. Of these regulators, IL-6, IL-1B, IFN-γ, IL-21, IL-5, IL-17A, TNF, IFN-A2 and IL-15 have been reported to regulate psoriatic inflammation [26]. Moreover, therapies targeting IL-1, IL-17 and TNF have shown promise in preclinical and clinical trials, implying that our analysis may provide insight into potential drug targets for psoriasis.

To further delineate critical regulatory networks in the pathogenesis of GPP, Regulatory Effects Analysis was performed using IPA. This analysis connects upstream regulators, DEGs in our dataset, and downstream functions or diseases to generate regulatory networks. A total of 69 Regulatory Effects networks were generated (*p* < 0.01). Remarkably, all the top 10 networks, ranked by consistency score, have downstream functions of “Activation of myeloid cells” and “Activation of phagocytes”. A regulatory network with the highest consistency score is shown in Fig. 4b. In this network, CSF1, F2RL1, FN1, IFN-γ, IL1R1, PI3K (complex), TLR2, TNF (family) and TRADD were identified as the upstream regulators, which target 24 downstream DEGs, resulting in an inhibitory effect on several immune cell types, including myeloid cells, phagocytes, and antigen presenting cells.

### Validation of RNA-Seq results with quantitative polymerase chain reactions (qPCR)

**Fig. 5:**
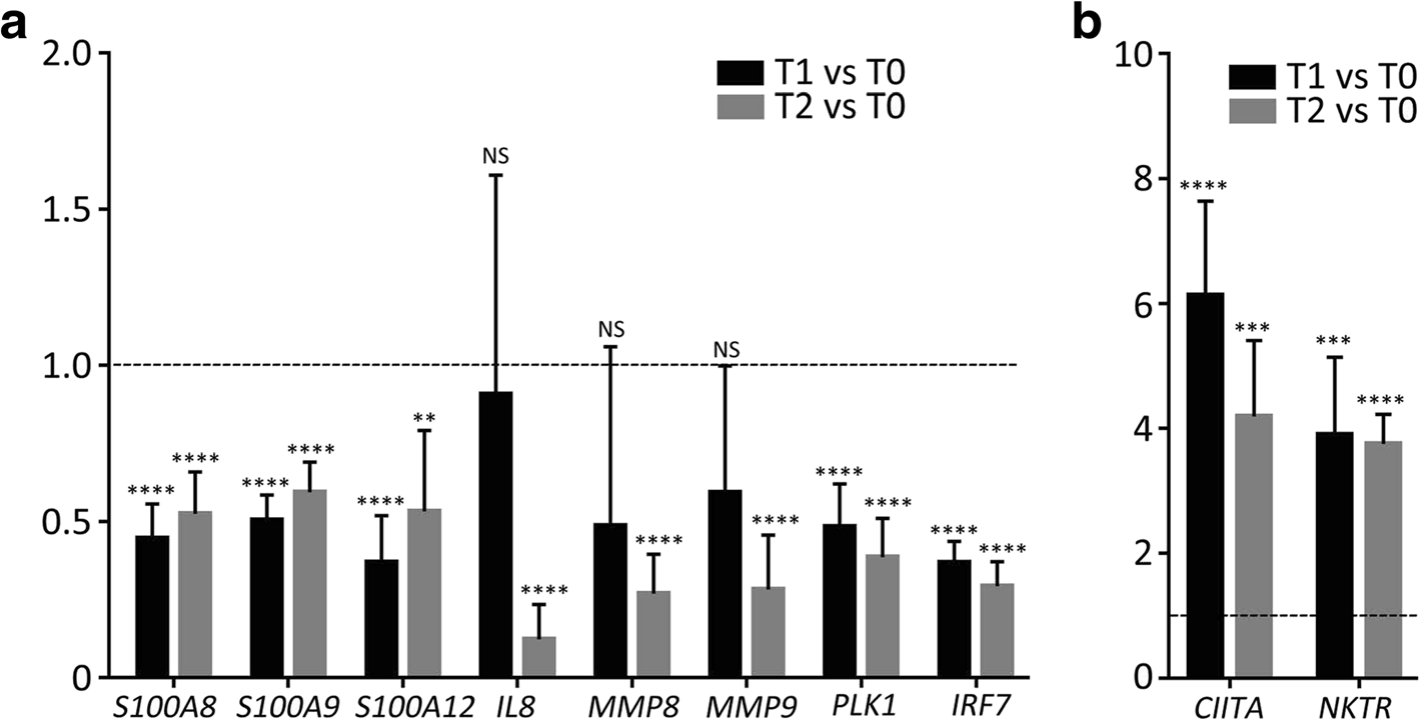
RT-qPCR validation of DEGs. The fold-change in expression is shown for **a** downregulated and **b** upregulated DEGs, in samples from T1 and T2, presented relative to the mean expression level of T0 samples, which is set at 1 in all cases. Error bars represent the standard error of the mean (NS, not significant, **p value ≤0.01, ***p value ≤0.001, ****p value ≤0.0001)

To verify the RNA-seq results, qPCR analyses were carried out for 10 genes (Fig. 5). Eight downregulated genes identified by the RNA-seq analysis were tested. Of these, *S100A8*, *S100A9*, *S100A12*, *IL-8*, *MMP8* and *MMP9* encode antimicrobial peptides (AMPs) or chemoattractant molecules previously known to be involved in GPP pathogenesis, and *PLK1* and *IRF7* were identified in the current research. In addition, two highly upregulated genes, *CIITA* and *NKTR* were chosen for validation. Using the 2^−ΔΔCt^ method, transcript levels for each gene were measured in comparison with the housekeeping gene, *GAPDH*, and were subsequently normalized to each value in the pre-treated (T0) sample. We observed significant increases in *CIITA* and *NKTR* expression, as well as significant decreases in *S100A8*, *S100A9*, *S100A12*, *PLK1* and *IRF7*, at both T1 and T2, which is similar to the RNA-seq data. Moreover, expression of *IL-8* and *MMP8* was found to significantly decrease at T2 but was not significantly changed at T1, which is also in agreement with the RNA-seq data. One exception is the *MMP9* gene: while a significant decrease was observed at both T1 and T2 in the RNA-seq analysis, a significant decrease was only observed at T2 in the qPCR experiment.

## Discussion

GPP is a severe skin disease associated with the eruption of sterile pustules and erythema. Pustular psoriasis lesions result from complex interactions among dermal or epidermal cells, resident and infiltrating immune cells, and a variety of cytokines. Previous studies have performed DEG analysis to investigate molecular abnormities in GPP patients [13, 14]. However, these studies focused on skin lesions, and the transcriptome signature of the peripheral circulation in GPP, a systematic autoinflammatory disease, remained largely unknown. In this study, we conducted a longitudinal RNA-seq-based transcriptome analysis of PBMCs from GPP patients before and after acitretin treatment. Our study revealed profound changes in the PBMC transcriptome, which may provide insights into GPP pathogenesis and potential biomarkers for diagnosis and therapeutics.

We found that DEGs were significantly enriched in neutrophil functions in our datasets. Neutrophils are commonly absent in the PBMC samples that are derived from healthy donors. However, a distinct subset of low-density granulocytes (LDGs) are present in PBMC preparations from patients with certain systemic inflammatory diseases [27]. For example, neutrophil-specific genes are abundant in the PBMCs from systemic lupus erythematosus (SLE) patients. This is due to the presence of LDGs in PBMCs from SLE patients [28, 29]. Besides, LDGs have also been identified in PBMCs from patients affected with psoriasis [30]. Thus, the enrichment of neutrophil-specific genes in our dataset may be resulted from elevated proportions of LDGs in GPP patients’ PBMCs. It is well-known that psoriasis is a T cell-, particularly a CD4^+^ and CD8^+^ T cell-mediated immune disease, but the function of neutrophils is much less well understood. Recent research has suggested that neutrophils may contribute to the pathogenic functions of IL-17, possibly in conjunction with the formation of neutrophil extracellular traps, and amplify psoriatic inflammation [30–32]. In our dataset, two of the most potent chemoattractant molecules for neutrophil recruitment, CXCL1 and CXCL8 (IL-8), were significantly downregulated in patients in remission from GPP. Moreover, DEGs were identified in nearly every aspect of neutrophil biology, including protein trafficking, granule formation, capture and rolling, pattern recognition and others (Table 2). Among these DEGs, two encoding the neutrophil G protein-coupled receptors, FPR1 and FPR2, which play pattern recognition roles in chemotaxis [33], were both downregulated. It is noteworthy that FPR2 is the receptor for LL-37, an AMP that drives psoriatic inflammation [34]. Other downregulated gene transcripts related to pattern recognition in neutrophils include *IFNGR1*, *LTB4R*, *TLR1*, *TLR5*, *MYD88*, *NLRP3* and *NLRC4*. The downregulation of these genes may negatively control AMP-mediated neutrophil activation, thus contributing to the resolution of inflammation in GPP patients. In addition to AMP receptors, genes encoding AMPs were also downregulated. These AMPs include LCN2, S100A8, S100A9 and S100A12. Although AMPs are believed largely to be produced by keratinocytes, neutrophil-released AMPs may contribute to the initiation of psoriasis [35]. Collectively, our results suggested that AMP–neutrophil interaction may play a role in the remission of GPP.

Recently, *AP1S3* mutations were found to be associated with pustular psoriasis, and knockdown of AP1S3 resulted in the upregulation of pro-inflammatory cytokines [7, 36]. AP1S3 is involved in protein trafficking in neutrophil activation [23]. In our datasets, *AP1S3* was significantly upregulated in post-treatment patients. Thus, it is tempting to speculate that the expression level of *AP1S3*, aside from its mutation, may also contribute to GPP pathogenesis by modulating the proinflammatory response.

PLK1 is a serine/threonine-protein kinase that triggers the G2/M transition of the cell cycle and performs important functions throughout the M phase [37]. IPA revealed that the “mitotic roles of PLK kinase” pathway was significantly enriched. Surprisingly, nearly all the pivotal players in this pathway, including *PLK1*, *CDC20*, *CDC25C*, *CYCLIN B2* and *MLKP1*, were downregulated in GPP patients upon acitretin treatment, suggesting a cell cycle-repressing effect of the drug. Although acitretin has been reported to have an anti-proliferative effect on hyper-proliferative tissues, such as psoriasis plaques [38], the mechanism of acitretin action in GPP remains largely elusive. In support of our observation, Liang [14] et al. reported that cell cycle checkpoint genes were significantly overexpressed in patients with GPP. In concert with its anti-proliferative effect on keratinocytes, acitretin may act to tune the cell cycle in the peripheral blood cells and contribute to quenching psoriatic inflammation.

Multiple cytokine signaling pathways involved in the pathogenesis of psoriasis, including IL-1, IL-6, IL-17A and interferon signaling, were enriched in the current analysis. These results are in agreement with a previous study of transcriptome profiles in pustular psoriatic lesions [14], suggesting that PBMCs and local skin cells may share similar patterns of immune dysregulation. IL-36 signaling is considered to be one of the pathological driving forces in GPP, and missense mutations in the IL-36 receptor antagonist were found to be the genetic cause of the disease in some patients [5]. Of note, in the current study, 3 out of 5 patients carry the c.115 + 6 T > C mutation in the *IL36RN* gene. More recently, a microarray analysis performed in psoriatic lesions from GPP patients suggested that the IL-1/IL-36 inflammatory axis appears to be central to the pathogenesis of GPP [13]. However, we did not detect changes in the transcripts of IL-36 cytokines in GPP patients under drug treatment. This discordance can be explained by the possibly distinct expression dynamics of IL-36 between blood and skin cells, or a lack of statistical power due to low sample numbers.

Upstream Regulator Analysis helps to identify critical nodes in a complex signaling network and provide potential drug targets. By this analysis, we identified multiple upstream regulators for a top-ranked network, including IL6, IL1B, IFNG, CSF2, IL21, IL17A, IFNA2, OSM and TNF, among others. TNF is known to play a central role in the inflammatory cytokine cascade, and TNF blockade in patients by biologics, such as infliximab, have shown success in GPP treatment [1]. Furthermore, IL17A is considered to be a druggable target for GPP, as evidenced by the effectiveness of IL17A blockade therapies [1]. Recently, inhibition of IL17A by secukinumab has been reported effective for a pediatric GPP patient with Deficiency of Interleukin-36-Receptor Antagonist (DITRA), implying a possible link between *IL36RN* mutation and Th17 differentiation in DITRA patients [39]. Moreover, we found that OSM, a cytokine secreted by skin-infiltrating T lymphocytes, was an upstream regulator of the network. OSM is a potent keratinocyte activator similar to TNF-α, IL-1, IL-17 and IL-22 [40]. Recently, OSM has been considered to be a drug target for multiple inflammatory diseases, such as colitis [41] and rheumatoid arthritis [42]. Based on the Upstream Regulator Analysis, we speculate that targeting the OSM pathway may have a beneficial effect on GPP.

Overall, our study provided a comprehensive gene expression profiling and molecular interaction network for GPP. GPP is likely to be genetically and physiopathologically heterogeneous among different patients. Thus, it would be beneficial to adopt specific immunointerventions for different cases. Combining the molecular profiling of a large cohort of clinical samples with in-depth bioinformatics analysis will provide further insights into strategies for GPP management and the development of tailored therapies.

## Conclusions

GPP is a fatal, multisystemic inflammatory disease. Our longitude analysis provided the first comprehensive study of transcriptome dynamics in the peripheral circulation of GPP patients and showed that the alleviation of GPP primarily involves the downregulation of leukocyte activity, particularly neutrophils. Moreover, the regulatory network constructed in the current research also provide a set of molecules with therapeutic relevance. Our study illustrates how RNA-seq-based transcriptomics can shed light on mechanism of disease and drug effects at the molecular level.

## Additional files

Additional file 1: **Table S1.** Primers used in RTqPCR validation. (DOCX 14 kb)

Additional file 2: **Table S2.** The top 50 upregulated and downregulated DEGs in the T1 versus T0 dataset. (DOCX 17 kb)

Additional file 3: **Table S3.** The top 50 upregulated and downregulated DEGs in the T2 versus T0 dataset. (DOCX 16 kb)

Additional file 4: **Figure S1.** Functional enrichment and annotation for DEGs in the “leukocyte activation involved in immune response” category. Enrichment of the top 20 clusters at T1 (panel A) and T2 (panel B) was performed using Metascape analysis. -log(FDR) values were calculated based on the accumulative hypergeometric distribution. (PDF 283 kb)

Additional file 5: **Figure S2.** Protein-protein interaction (PPI) enrichment analysis for DEGs in the “leukocyte activation involved in immune response” category. PPI analysis for the DEGs was first carried out using the BioGrid database. The Molecular Complex Detection (MCODE) algorithm was then employed to identify densely connected network components. Based on these two analyses, a PPI network was generated for DEGs at T1 (panel A) and T2 (panel B). Pathway and process enrichment analysis was applied to each MCODE component. The three (panel A) or four (panel B) best-scoring terms (by *p*-value) were retained as the functional description of the corresponding components. (PDF 231 kb)

Additional file 6: **Table S4.** List of DEGs enriched in canonical pathways by IPA analysis. (XLS 198 kb)

Additional file 7: **Figure S3.** Gene expression heatmaps for signaling pathways inhibited at both T1 and T2. Heatmaps of expression ratios and z-scores for the “TREM1 Signaling” (panel A), “Role of Pattern Recognition Receptors in Recognition of Bacteria and Viruses” (panel B) and“Interferon Signaling” (panel C) pathways are shown. The z-scores were calculated using the IPA z-score algorithm and predicted direction of change for the function. (PDF 167 kb)

Additional file 8: **Figure S4.** The expression ratios of innate immunity and adaptive immunity genes. Ratios were calculated with IPA My List Analysis and are presented as a bar chart. (PDF 85 kb)

Additional file 9: **Figure S5.** Gene expression heatmaps for DEGs enriched in cell cycle regulation and cytokine signaling pathways. Heatmaps of expression ratios and z-scores for the “Mitotic Roles of Polo-like Kinase” (panel A), “Cyclins and the Cell Cycle Regulation” (panel B) and “Role of IL-17A in Psoriasis” (panel C) pathways. The z-scores were calculated using the IPA z-score algorithm and predicted direction of change for the function. (PDF 152 kb)

## Abbreviations

AMP: Antimicrobial peptide
CORUM: Comprehensive Resource of Mammalian Protein Complexes
Ct: Threshold cycle
DEG: Differentially expressed gene
FDR: False discovery rate
GO: Gene ontology
GPP: Generalized pustular psoriasis
IPA: Ingenuity Pathway Analysis
KEGG: Kyoto Encyclopedia of Genes and Genomes
MCODE: Molecular Complex Detection
PBMC: Peripheral blood mononuclear cell
PCA: Principal component analysis
qPCR: Quantitative polymerase chain reaction
RNA-seq: RNA sequencing
RT-qPCR: Reverse transcription–quantitative polymerase chain reaction
