# Supplementary material for: RNA sequencing-based longitudinal transcriptomic profiling gives novel insights into the disease mechanism of generalized pustular psoriasis
Source: BMC Med Genomics. 2018 Jun 5;11:52. doi: 10.1186/s12920-018-0369-3 (PMC5989375; doi:10.1186/s12920-018-0369-3)
Supplement: Supplementary file 1 — Table S1. Primers used in RTqPCR validation. (DOCX 14 kb) [file 12920_2018_369_MOESM1_ESM.docx]

| Gene | Forward primer | Reverse primer |
| --- | --- | --- |
| *CIITA* | CCTGGAGCTTCTTAACAGCGA | TGTGTCGGGTTCTGAGTAGAG |
| *NKTR* | GAGCCGGTTGGTCGCATTAT | ACACGATGGAACGTAGAACCTT |
| *S100A8* | CATGCCGTCTACAGGGATGA | GACGTCTGCACCCTTTTTCC |
| *S100A9* | CCTGGACACAAATGCAGACAA | CGTCACCCTCGTGCATCTT |
| *S100A12* | AGCATCTGGAGGGAATTGTCA | GCAATGGCTACCAGGGATATGAA |
| *IL-8* | CAAGAGCCAGGAAGAAACCA | GTCCACTCTCAATCACTCTCAG |
| *MMP9* | GTGCTGGGCTGCTGCTTTGCTG | GTCGCCCTCAAAGGTTTGGAAT |
| *MMP8* | TGCTCTTACTCCATGTGCAGA | TCCAGGTAGTCCTGAACAGTTT |
| *PLK1* | AAAGAGATCCCGGAGGTCCTA | GGCTGCGGTGAATGGATATTTC |
| *IRF7* | GCTGGACGTGACCATCATGTA | GGGCCGTATAGGAACGTGC |

**Table S1** Primer sequence for quantitative real time reverse transcription-PCR
