# Supplementary material for: RNA sequencing-based longitudinal transcriptomic profiling gives novel insights into the disease mechanism of generalized pustular psoriasis
Source: BMC Med Genomics. 2018 Jun 5;11:52. doi: 10.1186/s12920-018-0369-3 (PMC5989375; doi:10.1186/s12920-018-0369-3)
Supplement: Supplementary file 2 — Table S2. The top 50 upregulated and downregulated DEGs in the T1 versus T0 dataset. (DOCX 17 kb) [file 12920_2018_369_MOESM2_ESM.docx]

**Table S2** Top 50 up-regulated and down-regulated genes（T1 vs T0）

| Up-regulated genes | logFC^a^ | FDR^b^ | Down-regulated genes | logFC | FDR |
| --- | --- | --- | --- | --- | --- |
| CIITA | 7.10 | 2.06E-07 | DDC8 | -6.21 | 6.35E-06 |
| DDX49 | 5.66 | 2.03E-06 | NIT1 | -5.34 | 6.55E-06 |
| GPR132 | 5.34 | 1.21E-05 | DCLRE1C | -5.76 | 9.03E-06 |
| ALG8 | 5.36 | 1.39E-05 | ST3GAL4 | -6.13 | 9.42E-06 |
| ACACA | 3.74 | 7.72E-05 | SDCCAG8 | -5.62 | 2.83E-05 |
| ENGASE | 5.22 | 1.48E-04 | TMEM128 | -4.14 | 1.01E-04 |
| TCTN3 | 3.71 | 2.19E-04 | CDC20 | -3.84 | 1.19E-04 |
| NIPSNAP1 | 4.31 | 2.29E-04 | IGFBP2 | -4.79 | 1.41E-04 |
| AGTPBP1 | 5.62 | 3.78E-04 | GK | -3.71 | 1.42E-04 |
| ACSF3 | 3.42 | 4.12E-04 | GOLGA4 | -4.02 | 1.43E-04 |
| EGF | 4.30 | 4.18E-04 | BCL6 | -1.90 | 3.29E-04 |
| KLHL7 | 5.76 | 4.51E-04 | IL1R2 | -3.55 | 3.67E-04 |
| TBKBP1 | 4.28 | 5.10E-04 | KCNN4 | -3.02 | 4.37E-04 |
| HNRNPL | 4.36 | 5.23E-04 | SYNE1 | -5.78 | 4.86E-04 |
| PIH1D1 | 3.18 | 5.54E-04 | PRKAG2 | -2.25 | 5.00E-04 |
| ZNF420 | 3.70 | 5.57E-04 | DYSF | -1.89 | 5.55E-04 |
| ADCY7 | 1.24 | 5.92E-04 | WDFY3 | -1.74 | 5.58E-04 |
| RPS27 | 1.42 | 6.81E-04 | ASPM | -2.20 | 5.62E-04 |
| CD22 | 1.43 | 7.10E-04 | F5 | -1.41 | 6.06E-04 |
| BZRAP1 | 6.25 | 7.49E-04 | STAB1 | -1.05 | 6.19E-04 |
| FAM107B | 7.17 | 7.62E-04 | CES1 | -1.41 | 6.22E-04 |
| LTBP3 | 2.36 | 7.88E-04 | PKN2 | -6.17 | 6.24E-04 |
| IGHD | 1.65 | 8.22E-04 | KIAA0040 | -5.52 | 6.49E-04 |
| FAM193B | 5.36 | 8.46E-04 | SNX32 | -1.36 | 6.52E-04 |
| WDR44 | 3.35 | 1.01E-03 | CHD1L | -3.99 | 6.83E-04 |
| WWP2 | 3.26 | 1.01E-03 | DHX38 | -4.89 | 6.85E-04 |
| THOC7 | 4.30 | 1.03E-03 | ZFP64 | -5.09 | 7.08E-04 |
| DBNDD2 | 3.27 | 1.03E-03 | FCGR1A | -3.12 | 7.23E-04 |
| FAM102A | 5.03 | 1.04E-03 | FLVCR2 | -1.22 | 7.97E-04 |
| STRBP | 1.09 | 1.06E-03 | MAPK14 | -1.14 | 8.04E-04 |
| FAM129C | 1.48 | 1.09E-03 | ATG3 | -4.26 | 8.25E-04 |
| MEMO1 | 4.45 | 1.15E-03 | ACTB | -1.31 | 8.56E-04 |
| NOC3L | 4.74 | 1.23E-03 | GNAS | -4.12 | 8.59E-04 |
| RNF170 | 3.63 | 1.32E-03 | PDLIM7 | -4.06 | 8.87E-04 |
| PPP2R4 | 4.50 | 1.37E-03 | FGD4 | -1.34 | 9.35E-04 |
| HERC1 | 3.68 | 1.50E-03 | KCNN3 | -3.83 | 9.65E-04 |
| GIGYF2 | 4.26 | 1.56E-03 | ST3GAL3 | -3.34 | 9.69E-04 |
| MS4A1 | 1.05 | 1.58E-03 | PALB2 | -5.11 | 9.87E-04 |
| SCFD2 | 4.37 | 1.68E-03 | WDFY3 | -1.64 | 1.00E-03 |
| RARG | 4.85 | 1.71E-03 | FCGRT | -5.67 | 1.01E-03 |
| FCRL1 | 1.03 | 1.79E-03 | APOBEC3A | -4.75 | 1.03E-03 |
| RCOR3 | 4.52 | 1.83E-03 | CDCA3 | -2.81 | 1.06E-03 |
| **Table S2** Top 50 differentially expressed genes（T1 vs T0）(*Continued*) | | | | | |
| PLD4 | 1.47 | 1.94E-03 | OAZ1 | -2.02 | 1.07E-03 |
| PIK3R5 | 5.26 | 1.98E-03 | DOCK8 | -6.68 | 1.08E-03 |
| INTS2 | 3.95 | 2.06E-03 | LRRFIP1 | -2.02 | 1.17E-03 |
| CTD-2192J16.22 | 2.68 | 2.13E-03 | CD55 | -1.15 | 1.18E-03 |
| PRDM2 | 1.18 | 2.17E-03 | CREB5 | -1.76 | 1.22E-03 |
| RTCB | 4.20 | 2.24E-03 | TMEM184B | -5.17 | 1.23E-03 |
| TRAPPC11 | 4.61 | 2.24E-03 | GIGYF2 | -3.81 | 1.24E-03 |
| RNF126 | 4.15 | 2.24E-03 | HJURP | -4.91 | 1.24E-03 |

^a^LogFC: fold change expressed as log base 2

^b^FDR: p value adjusted using Benjamini Hochberg method
