# Supplementary material for: RNA sequencing-based longitudinal transcriptomic profiling gives novel insights into the disease mechanism of generalized pustular psoriasis
Source: BMC Med Genomics. 2018 Jun 5;11:52. doi: 10.1186/s12920-018-0369-3 (PMC5989375; doi:10.1186/s12920-018-0369-3)
Supplement: Supplementary file 3 — Table S3. The top 50 upregulated and downregulated DEGs in the T2 versus T0 dataset. (DOCX 16 kb) [file 12920_2018_369_MOESM3_ESM.docx]

**Table S3** Top 50 up-regulated and down-regulated genes（T2 vs T0）

| Up-regulated genes | logFC^a^ | FDR^b^ | Down-regulated genes | logFC | FDR |
| --- | --- | --- | --- | --- | --- |
| YIF1A | 4.21 | 2.48E-05 | ZNF185 | -6.55 | 1.56E-06 |
| ATP1B1 | 4.75 | 3.61E-05 | ENTHD2 | -5.77 | 3.21E-05 |
| RPS6KA4 | 6.56 | 8.73E-05 | ZNF557 | -5.97 | 4.84E-05 |
| HSD17B11 | 3.91 | 1.10E-04 | RREB1 | -5.53 | 5.25E-05 |
| FCRL1 | 1.11 | 1.22E-04 | ODF3B | -4.37 | 6.02E-05 |
| B3GALNT2 | 6.35 | 1.37E-04 | HBB | -2.64 | 6.83E-05 |
| RPL3 | 1.37 | 1.87E-04 | TPM4 | -1.70 | 7.89E-05 |
| ZSCAN9 | 4.24 | 2.38E-04 | DNAJB4 | -3.83 | 9.67E-05 |
| RPL3 | 1.00 | 3.23E-04 | PSMB5 | -4.77 | 1.06E-04 |
| NAMPT | 1.73 | 3.40E-04 | RNF10 | -4.95 | 1.15E-04 |
| AC004381.6 | 3.10 | 4.80E-04 | SLC4A10 | -6.77 | 1.43E-04 |
| PPP6R3 | 4.69 | 6.29E-04 | DENND1B | -6.52 | 1.44E-04 |
| PBX4 | 3.63 | 6.73E-04 | PRKCD | -3.49 | 1.67E-04 |
| ATR | 5.06 | 6.75E-04 | IGHG1 | -3.61 | 1.70E-04 |
| PRUNE | 5.71 | 7.12E-04 | CCDC90B | -3.50 | 1.76E-04 |
| BZRAP1 | 6.29 | 7.33E-04 | MLKL | -5.23 | 2.10E-04 |
| ERCC2 | 4.46 | 7.33E-04 | LPAR2 | -5.55 | 2.17E-04 |
| METTL22 | 4.59 | 7.74E-04 | SOCS3 | -2.03 | 2.25E-04 |
| SEC24C | 7.08 | 7.78E-04 | SLC11A1 | -1.22 | 2.34E-04 |
| KRI1 | 3.46 | 8.05E-04 | CDC20 | -5.34 | 2.64E-04 |
| SMIM7 | 4.01 | 8.96E-04 | EGR1 | -1.77 | 3.01E-04 |
| C2CD3 | 4.97 | 8.98E-04 | VMP1 | -1.48 | 3.50E-04 |
| DHX15 | 5.64 | 9.23E-04 | PSAP | -1.23 | 3.59E-04 |
| FAM101B | 1.70 | 9.36E-04 | KCNN3 | -4.36 | 3.74E-04 |
| SLC4A4 | 4.13 | 9.55E-04 | CR1 | -1.78 | 5.39E-04 |
| EPHB1 | 3.13 | 1.06E-03 | DDX60L | -7.97 | 5.84E-04 |
| C9orf85 | 2.97 | 1.11E-03 | ASPM | -2.09 | 5.87E-04 |
| RPL14 | 5.11 | 1.12E-03 | FAXDC2 | -5.75 | 5.88E-04 |
| RPL7A | 3.94 | 1.14E-03 | CSAD | -3.06 | 5.88E-04 |
| EOMES | 3.57 | 1.15E-03 | SLC2A3 | -1.32 | 6.53E-04 |
| FAM129C | 1.25 | 1.16E-03 | PTEN | -8.23 | 6.58E-04 |
| ARL6IP1 | 4.73 | 1.25E-03 | NFXL1 | -5.61 | 6.98E-04 |
| CIRBP | 5.76 | 1.32E-03 | GAS6 | -2.15 | 7.08E-04 |
| HADH | 3.47 | 1.39E-03 | TOP2A | -1.91 | 7.11E-04 |
| SLC4A7 | 5.13 | 1.39E-03 | S100A9 | -1.26 | 7.18E-04 |
| MKLN1 | 4.38 | 1.40E-03 | E2F2 | -1.81 | 7.19E-04 |
| C8orf59 | 4.29 | 1.41E-03 | ZNF148 | -5.03 | 7.22E-04 |
| CD79B | 1.20 | 1.49E-03 | NFAT5 | -5.79 | 7.38E-04 |
| TGFBR1 | 1.58 | 1.54E-03 | MAP7D1 | -4.91 | 7.92E-04 |
| RPSA | 5.41 | 1.65E-03 | SPTB | -6.66 | 8.07E-04 |
| PPP3CB | 2.44 | 1.66E-03 | PLSCR1 | -2.03 | 8.27E-04 |
| USP28 | 5.58 | 1.67E-03 | ZNF83 | -6.35 | 8.37E-04 |
| **Table S3** Top 50 up-regulated and down-regulated genes（T2 vs T0）(*Continued*) | | | | | |
| SPINT2 | 4.30 | 1.74E-03 | OTUD1 | -1.05 | 8.41E-04 |
| ISPD | 4.00 | 1.74E-03 | DYSF | -1.32 | 8.45E-04 |
| GOLGB1 | 1.13 | 1.78E-03 | PLBD1 | -1.05 | 8.50E-04 |
| PITPNM2 | 4.29 | 1.80E-03 | MEF2BNB | -4.49 | 8.71E-04 |
| CD22 | 1.15 | 1.89E-03 | PHACTR2 | -1.16 | 8.76E-04 |
| DCAF8 | 2.93 | 2.02E-03 | IRF7 | -5.45 | 8.78E-04 |
| MCEE | 4.15 | 2.03E-03 | GSPT1 | -5.38 | 8.87E-04 |
| HSP90AB1 | 1.14 | 2.05E-03 | DDC8 | -5.58 | 9.34E-04 |

^a^LogFC: fold change expressed as log base 2

^b^FDR: p value adjusted using Benjamini Hochberg method
