# Supplementary figures and images for: RNA sequencing-based longitudinal transcriptomic profiling gives novel insights into the disease mechanism of generalized pustular psoriasis
Source: BMC Med Genomics. 2018 Jun 5;11:52. doi: 10.1186/s12920-018-0369-3 (PMC5989375; doi:10.1186/s12920-018-0369-3)

A

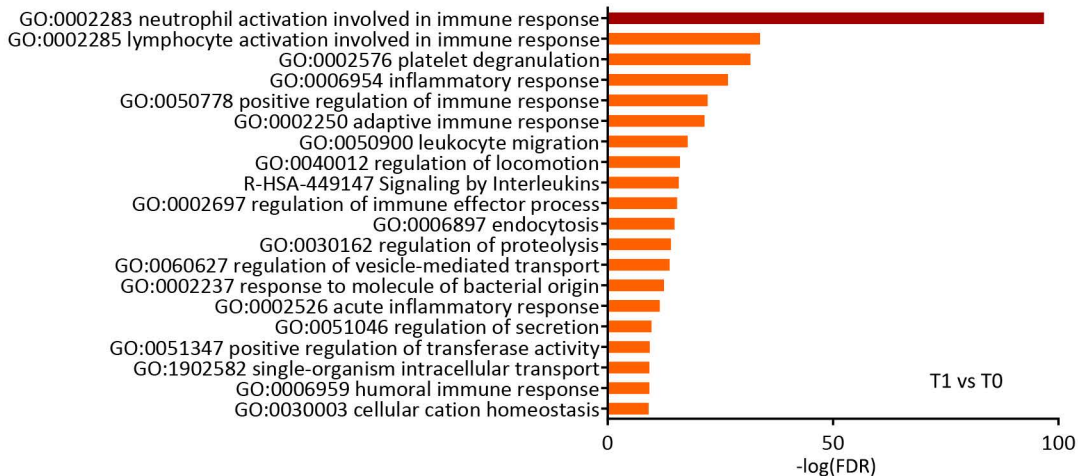

B

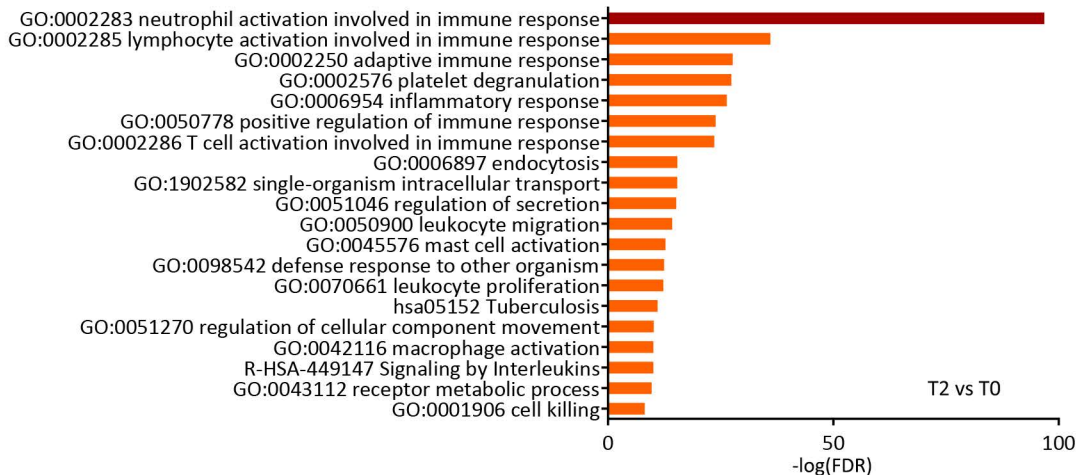

Supplement: Supplementary file 4 — Figure S1. Functional enrichment and annotation for DEGs in the “leukocyte activation involved in immune response” category. Enrichment of the top 20 clusters at T1 (panel A) and T2 (panel B) was performed using Metascape analysis. -log(FDR) values were calculated based on the accumulative hypergeometric distribution. (PDF 283 kb) [file 12920_2018_369_MOESM4_ESM.pdf]

A

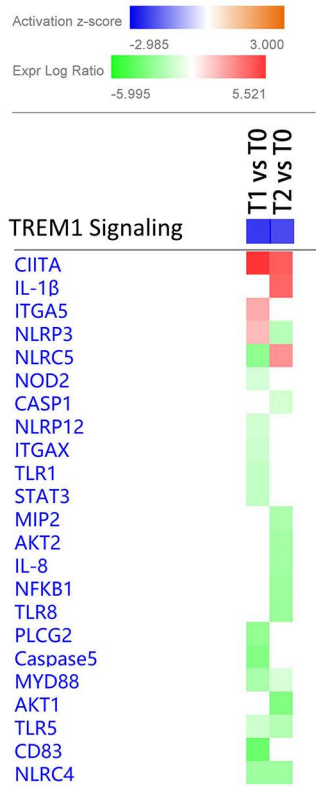

B

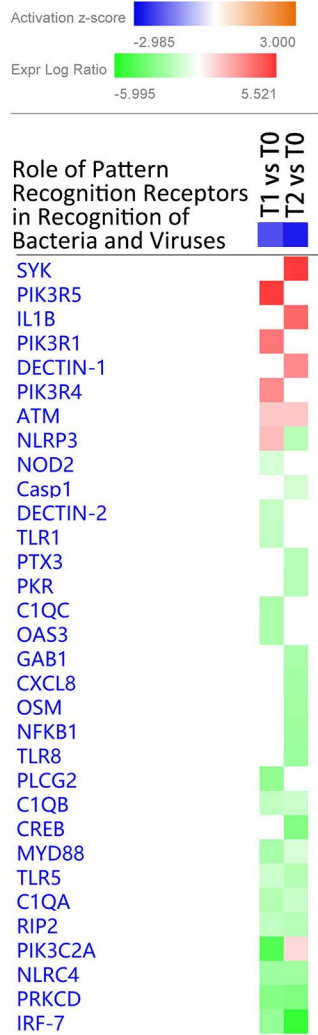

C

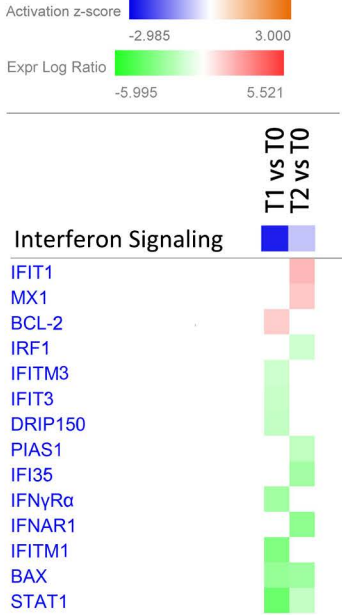

Supplement: Supplementary file 7 — Figure S3. Gene expression heatmaps for signaling pathways inhibited at both T1 and T2. Heatmaps of expression ratios and z-scores for the “TREM1 Signaling” (panel A), “Role of Pattern Recognition Receptors in Recognition of Bacteria and Viruses” (panel B) and“Interferon Signaling” (panel C) pathways are shown. The z-scores were calculated using the IPA z-score algorithm and predicted direction of change for the function. (PDF 167 kb) [file 12920_2018_369_MOESM7_ESM.pdf]

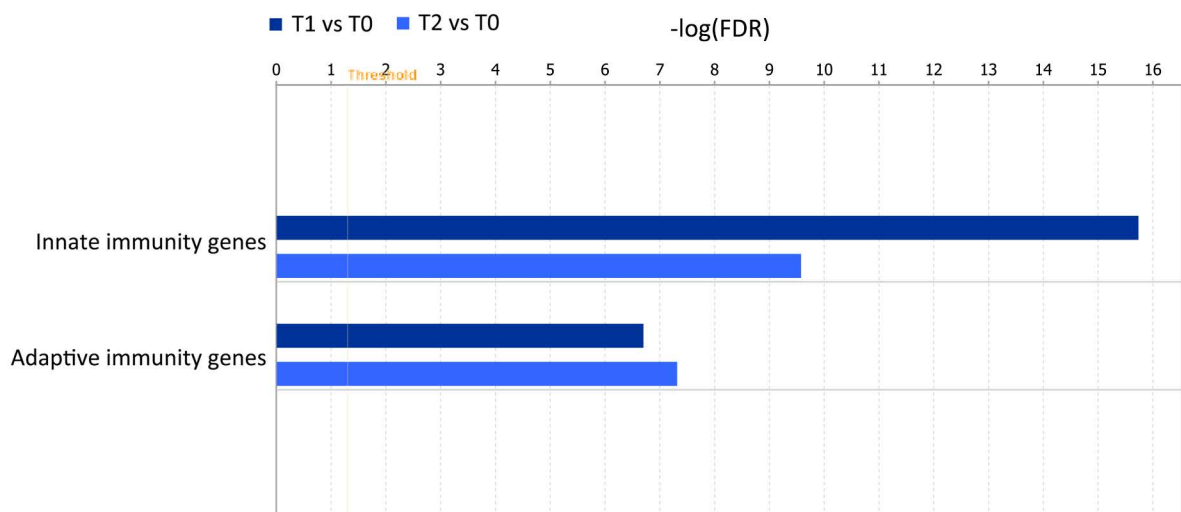

Supplement: Supplementary file 8 — Figure S4. The expression ratios of innate immunity and adaptive immunity genes. Ratios were calculated with IPA My List Analysis and are presented as a bar chart. (PDF 85 kb) [file 12920_2018_369_MOESM8_ESM.pdf]

A

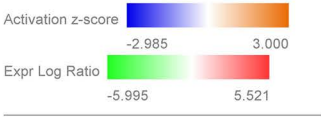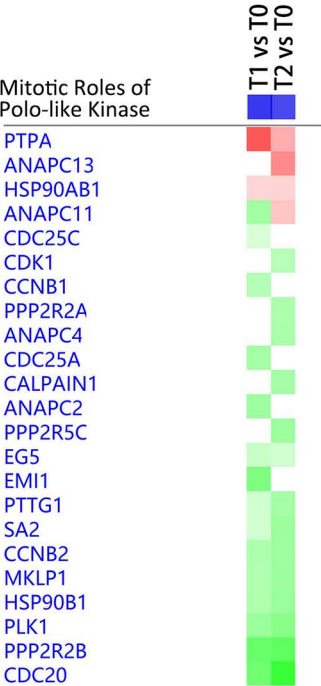

B

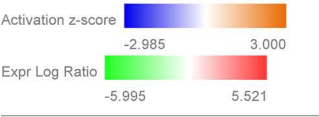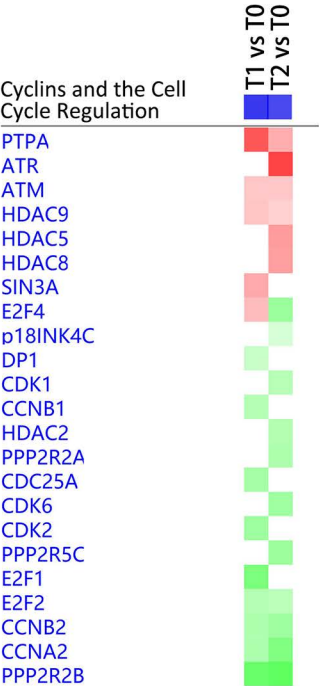

C

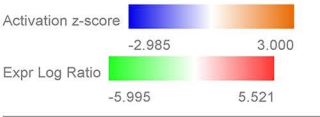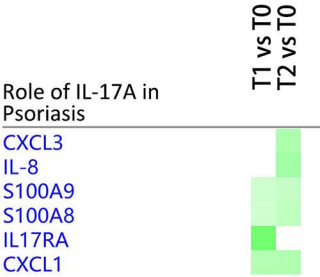

Supplement: Supplementary file 9 — Figure S5. Gene expression heatmaps for DEGs enriched in cell cycle regulation and cytokine signaling pathways. Heatmaps of expression ratios and z-scores for the “Mitotic Roles of Polo-like Kinase” (panel A), “Cyclins and the Cell Cycle Regulation” (panel B) and “Role of IL-17A in Psoriasis” (panel C) pathways. The z-scores were calculated using the IPA z-score algorithm and predicted direction of change for the function. (PDF 152 kb) [file 12920_2018_369_MOESM9_ESM.pdf]
