# Supplementary material for: RNA sequencing-based longitudinal transcriptomic profiling gives novel insights into the disease mechanism of generalized pustular psoriasis
Source: BMC Med Genomics. 2018 Jun 5;11:52. doi: 10.1186/s12920-018-0369-3 (PMC5989375; doi:10.1186/s12920-018-0369-3)
Supplement: Supplementary file 5 — Figure S2. Protein-protein interaction (PPI) enrichment analysis for DEGs in the “leukocyte activation involved in immune response” category. PPI analysis for the DEGs was first carried out using the BioGrid database. The Molecular Complex Detection (MCODE) algorithm was then employed to identify densely connected network components. Based on these two analyses, a PPI network was generated for DEGs at T1 (panel A) and T2 (panel B). Pathway and process enrichment analysis was applied to each MCODE component. The three (panel A) or four (panel B) best-scoring terms (by p-value) were retained as the functional description of the corresponding components. (PDF 231 kb) [file 12920_2018_369_MOESM5_ESM.pdf]

A

T1 vs T0

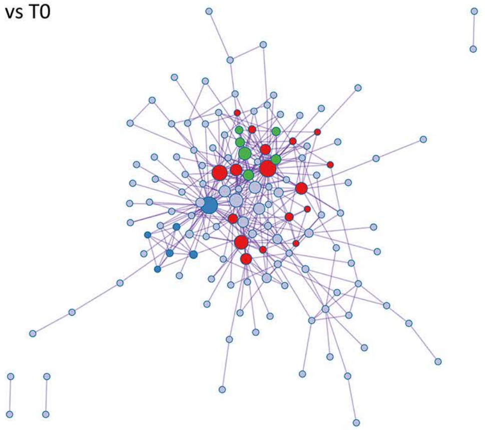

Color	MCODE	GO	Description	Log10(P)
	MCODE_1	GO:0045055	regulated exocytosis	-20.1
	MCODE_1	GO:0002366	leukocyte activation involved in immune response	-14.5
	MCODE_1	GO:0002263	cell activation involved in immune response	-14.5
	MCODE_2	R-HSA-444473	Formyl peptide receptors bind formyl peptides and many other ligands	-13.0
	MCODE_2	R-HSA-375276	Peptide ligand-binding receptors	-12.5
	MCODE_2	R-HSA-373076	Class A/1 (Rhodopsin-like receptors)	-11.0
	MCODE_3	R-HSA-6798695	Neutrophil degranulation	-10.0
	MCODE_3	GO:0043312	neutrophil degranulation	-10.0
	MCODE_3	GO:0002283	neutrophil activation involved in immune response	-10.0

B

T2 vs T0

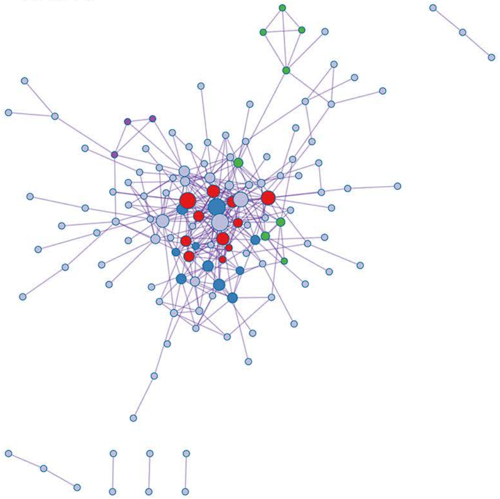

Color	MCODE	GO	Description	Log10(P)
	MCODE_1	GO:0045055	regulated exocytosis	-16.3
	MCODE_1	R-HSA-354192	Integrin alphaIIb beta3 signaling	-15.1
	MCODE_1	R-HSA-9006921	Integrin signaling	-15.1
	MCODE_2	GO:0045055	regulated exocytosis	-12.3
	MCODE_2	GO:0002274	myeloid leukocyte activation	-8.9
	MCODE_2	GO:0002366	leukocyte activation involved in immune response	-8.6
	MCODE_3	GO:0002274	myeloid leukocyte activation	-10.0
	MCODE_3	GO:0042119	neutrophil activation	-8.5
	MCODE_3	GO:0036230	granulocyte activation	-8.5
	MCODE_4	GO:0002755	MyD88-dependent toll-like receptor signaling pathway	-8.4
	MCODE_4	R-HSA-975138	TRAF6 mediated induction of NFkB and MAP kinases upon TLR7/8 or 9 activation	-7.2
	MCODE_4	R-HSA-168181	Toll Like Receptor 7/8 (TLR7/8) Cascade	-7.1
